# Information-Based Plastic Material Tracking for Circular Economy—A Review

**DOI:** 10.3390/polym15071623

**Published:** 2023-03-24

**Authors:** Thomas Rumetshofer, Jörg Fischer

**Affiliations:** Institute of Polymeric Materials and Testing, Johannes Kepler University Linz, Altenberger Straße 69, 4040 Linz, Austria

**Keywords:** plastic material, traceability, circular economy, physical tracking, blockchain, digital product passport, certification

## Abstract

At the moment, it looks like the plastics recycling industry is skimming only low-hanging fruits of its business. To reach intended targets, a greater effort and disruptive innovations are necessary. Physical- or digital-information-based solutions for tracking plastic material can support the circular economy and help to overcome hurdles along the value chain. In this paper, the scientific literature and initiatives in four different technology areas for information-based tracking solutions are reviewed and analyzed. Physical markers can improve sorting efficiencies on short notice but adhere some technical difficulties. Blockchain as a new concept promises high transparency and security, with the drawbacks of energy-intense verification and technical uncertainties. As a third group, the digital product passport claims a combination of physical and digital solutions with open questions on data ownership. The fourth and last group includes standards and certification systems that aim for maximum consensus with slow market implementation. To enable an integrated circular economy of plastics, plastic material tracking solutions must experience broad acceptance by all players along the value chain in the plastics industry and they should additionally be supported by society.

## 1. Introduction

Plastics are very versatile materials used in many different applications in everyday life all over the world, with substantial benefits such as durability, flexibility, low weight, and lower cost compared to other alternatives [1,2]. Global plastics production in 2020 without recycled plastics was 367 million tons, with a share of 55 million tons attributed to Europe [3]. The majority of these materials are fossil-based and contribute to a linear economy.

This linear economy of plastics, consisting of produce-use-dispose, creates problems such as the leaking of materials into the environment and the emitting of CO2 through incineration. Every year, an estimated amount of 4.8 to 12.7 million tons of plastics enters the ocean from coastal countries [4]. In 2020, an amount of 29.5 million tons of post-consumer plastic waste (PCW) was collected within Europe. From this PCW stream, 23.4% was landfilled, 42.0% went into incineration, and 34.6% passed toward recycling [3]. The gap of approximately 25.5 million tons between plastic production and plastic waste collection comes mainly from the different lifespan of products leading to the accumulation of plastic products, which reflects its future potential as feedstock for recycling.

One proposed solution to overcome the above-mentioned hurdles connected to the use of plastics in a linear economy is a transformation into a circular economy to keep materials in the loop and reduce the consumption of fossil resources [5]. Circular economy models are inspired by natural processes and can be applied in many areas. There is no archetypal form of circular economy available that enables the solution of every problem. The prevention of waste, optimized material flow, maximized resource recovery, and energy-from-waste are a few advantages of circular economy models [6]. In the literature, more than 30 different definitions of “circular economy” are known, closely related to resource and environmental concepts [7]. 

Politicians from the European Commission have also realized problems around plastics and published in 2015 an action plan for a circular economy dealing with marine litter, hazardous chemical additives, and the recycling of plastic packaging [8]. Based on this action plan, a European strategy for plastics was brought on the way in 2018 as a framework for driving innovation, investments, and circular solutions [9]. The aim of this strategy is to improve economics and plastics recycling to turn current challenges into opportunities for Europe.

In a recently published action plan, the European Commission wants to tackle problems along the entire life-cycle of a product with measures in many different sectors. For plastic products, a phase-out of single-use plastics (SUPs) and a restriction of intentionally added microplastics are mentioned as actions [10].

Around these European strategy and action plans, a number of directives are created or amended to ensure implementation:

- Packaging and packaging waste directive (94/62/EG) aims for harmonizing the management of packaging and packaging waste within the European Union with its amendment in 2018 [11]. In this directive, the hierarchy of waste (reduce, reuse, recycle, dispose) is established and the latest targets on the collection of packaging are included. For plastic packaging, this means a recycling rate of at least 50% by the end of 2025 and 55% by the end of 2030.
- European directive (EU/2015/720) to reduce the consumption of lightweight plastic carrier bags to prevent the littering of plastic carrier bags and other items into the environment [12].
- European directive (EU/2019/904) to reduce the impact of certain plastic products on the environment. This directive restricts the consumption of SUP items within Europe [13].
- Regulation on shipment of waste (EC/1013/2006), with an amendment to the Basel Convention in 2020, has the primary objective of protecting the environment in international trade [14,15].

**Figure 1:**
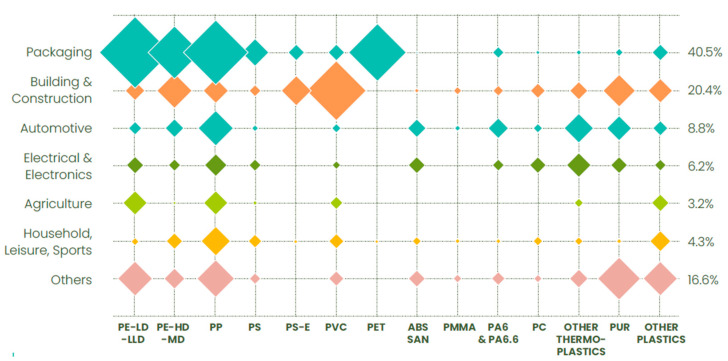
European plastic demand by segment and polymer [3].

Today’s most commonly used technology to keep polymeric materials in the loop is mechanical recycling, which usually includes waste collection, sorting, grinding, washing, and extrusion [16]. PCW streams can come from multiple sources and applications. The largest share of all plastics produced goes into packing applications (40.5%), as can be seen in Figure 1 [3]. Moreover, plastics in packaging applications have with less than 1 year a very short lifespan compared to other applications [17], which leads to a high volume of packaging plastics in mechanical recycling facilities. Packaging polymer materials include mainly polyethylene low-density (PE-LD), polyethylene high-density (PE-HD), polypropylene (PP), and polyethylene terephthalate (PET). While the polyolefins PE-LD, PE-HD, and PP are utilized in flexible and rigid packaging applications as well as in other application areas, PET is mainly used in packaging for rigid bottle and tray applications and has an overall polymer share of 8.4% in Europe [3].

In 2020, the plastics recycling industry in Europe counted 600 companies with a total installed capacity of 8.5 million tons [18]. Only 5 European countries (DE, NED, UK, IT, SPA) cover 2/3 of this capacity. Interestingly, PET has a share of 30% of the total installed recycling capacity in Europe [18]. This is over-proportional compared to the virgin PET production of 8.4% and can be explained by polymer type properties, the maturity of the recycling process, and the limited number of applications [19].

## 2. Complexity in Plastics Recycling and Scope

**Figure 2:**
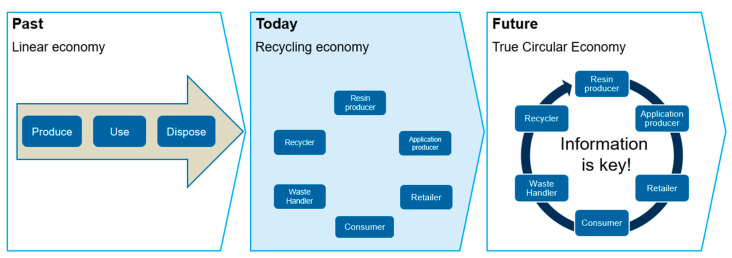
Business models of plastics industry for past, present, and future.

Figure 2 illustrates business models for the past, current, and future plastics industry. In the past, a linear economy model consisting of make, use, and dispose was favored, whereas, nowadays, the preferred model reflects a circular economy and consists of the connection and interaction of all players along the plastic value chain (VC). The main obstacles in today’s recycling model are the lack of information sharing between all players along the VC and its increasing complexity.

One side of the increasing complexity and pressure for the plastics industry comes from European legislation through increased recycling targets, as mentioned in the section above. Simultaneously, clear guidance and definitions around recyclability, recycling, recycling technologies, and quality criteria are missing [20]. For example, food contact material (FCM) obtained from recyclates is a legally very complex topic in European law [21].

In some cases, the use of alternative materials instead of fossil-based plastics is realized without considering implications for all players along the value chain. Alternative materials can be bio-degradable polymers, paper, glass, cotton, and others. As consumers, waste management systems, and recycling systems are not suited for these materials, these material changes create additional irritation and complexity within usage, recycling, and end-of-life solutions [22]. It is essential to emphasize that alternative material solutions very often arise only as a result of trends and do not always bring ecological or economic advantages. For example, a recent study on plastic carrier bags and their alternatives showed an environmental benefit of at least 50% for the plastic-based solutions compared to the alternatives in the evaluated scenarios [23]. As another example, the deployment of carbon-only backbone polymers, containing pro-oxidant additives, can help to reduce the breakdown time of littered plastics, with the downside of irritation in the circular economy [24].

A further reason to enhance the complexity of plastics recycling is the high variability in material type and composition. Multilayer and composite materials, often not designed to be recyclable in established recycling streams, lead to more efforts in separation and recycling operations. Furthermore, already recycled material enters the plastic value chain in large volumes, along with other newly established routes such as bio-based, chemically recycled, or bio-degradable materials. For example, wood-plastic composites (WPCs), which contain up to 80% wood fibers, are now entering packaging applications [25]. The high filler content of these material solutions often has a negative impact on the recycling rates or the property profiles of the recyclates. In addition, colored plastics can influence recycling processes. In particular, carbon-black-colored polymers, which are not detectable by state-of-the-art sorting systems, pose a problem. They represent a large volume in automotive and electronic product applications [26]. These materials are lost for further recycling.

Engineers around the globe try to solve the problems around plastics recycling with the development of new technologies and production routes in the areas of gasification [16], solvent-based recycling [27], or chemical recycling [28]. This leads to a more complex landscape of possible technologies and products in the future. 

On top of all the complexity that comes from legislation, materials, processes, and technologies, plastics recycling lacks comprehensive data management. Data on processes and products are generated but only used in the company’s own isolated premises, and are not shared with others [29].

A future business model needs to be able to transform the plastics industry into a true circular economy. Therefore, many changes are required, starting with the feedstock, the energy supply, and the collection system. A very important aspect is traceability and information exchange between all partners in the future value chain of the plastics industry. This solution can help overcome current hurdles and complexities. This paper aims to provide an overview of the current state of academic work and initiatives to support traceability in the plastics industry. The focus is on material tracking along the value chain in plastics recycling. To this end, four different technology areas for information-based tracking solutions are reviewed and analyzed.

## 3. Tracking Solutions for Plastics

In the context of this paper, tracking means the consistent following of material and information from feedstock to final product and beyond. Tracking without disruption is already established in some food supply chain areas to ensure food safety and quality without commercial fraud [30].

A reliable and consistent tracking system is always linked to additional efforts and costs along the value chain. On the other hand, for some applications, traceability is a prerequisite for a circular economy or can lead to a competitive advantage. Closed-loop systems, for example, require a certain knowledge on the origin and history of the material used. Mechanical recycling for FCM needs a defined amount of material previously used in food contact applications and the resulting recyclates should be free of harmful substances predetermined by the FDA or EFSA [21]. Consistently, the traceability of material and origin is inevitable if the carbon tax proposed by the European Commission or environmentally modulated taxes become a reality [31]. In all these cases, consistent and intelligent tracking can support bringing plastic products with a competitive advantage to the market. In addition, technological advances in physical and chemical tracking can contribute to circular economy ambitions [32].

A sustainable business model for plastics is defined as minimizing environmental damage through improved waste management, recovery of plastics from the environment, or reuse of plastics [33]. Traceability can support enhancing existing business models to make them more sustainable and it opens possibilities for different business models going along with the plastic value chain.

For the enhancement of an existing business model toward a more sustainable or circular business model, an additional value in one of the four following categories needs to be added [29]:

- Generation of additional revenue through increased sales or market share generated by sustainability.
- Sustainability enhancement of the own brand for employee engagement or to attract investors.
- Cost savings from lower consumption of material or energy.
- Risk reduction by increased reputation or decreased regulatory risk.

Besides improvements on already established businesses, consistent traceability can offer possibilities for a completely new set of business models linked to the circular economy of plastics [29]:

- New products by circular inputs, including the use of bio-based or completely recyclable materials and the rethinking of product designs.
- Data sharing platforms that provide access to data about products or materials.
- Product as a service. Complete change in product ownership combined with a lease or rent model for products and resources.
- Recovery of all forms of resources, by-products, and energy.

**Figure 3:**
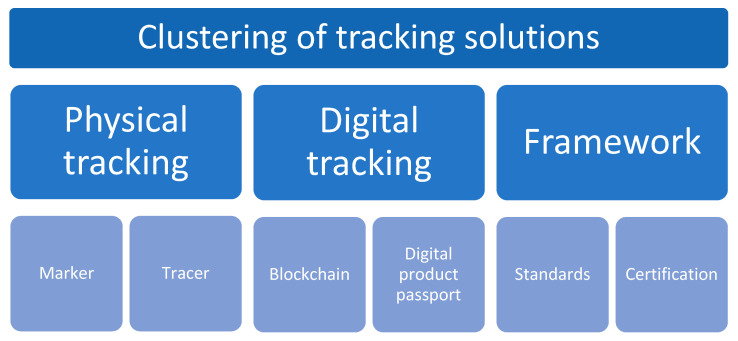
Clustering of tracking solutions.

Traceability in the plastics industry can take a variety of forms, ranging from physical marking to a purely digital value. In many cases, it is necessary to combine a physical marking of materials and products with digital data to ensure uninterrupted information sharing. Figure 3 illustrates an overview of the different solutions for tracking in the plastics industry. In general, the various solutions can be divided into four categories:

- Marker or tracer for plastics.
- Blockchain for plastics.
- Digital product passport for plastics.
- Certification systems for plastics.

### 3.1. Marker or Tracer for Plastics

**Figure 4:**
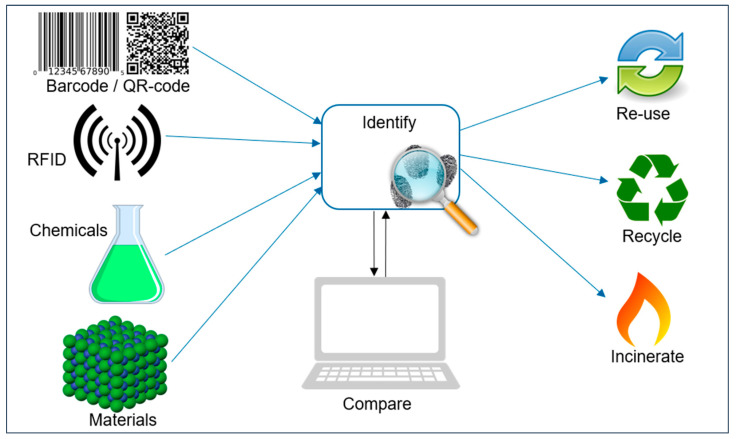
Options for physical tracking options in plastics recycling; Graphics from [37].

Physical tracking can be realized by an optical identifier (marker) or by the addition of material to the polymer matrix or surface (tracer). Information needs to be retrieved from a marker or tracer, identified, and compared with a database in order to decide how the lifetime of the plastic material can be further prolonged or treated. Figure 4 shows options for physical tracking using marker or tracer technologies.

In the literature, a high number of scientific publications can be found on markers and tracers for plastics, and interest has increased in recent years, exceeding 200 publications per year in 2016. A Scopus search on ((plastic OR plastics) AND (tracking OR marker OR tracer)) delivered 4802 hits when results were limited to engineering, chemistry, and material science. Table 1 summarizes the most relevant papers and initiatives relevant for physical tracking in the plastics industry.

The simplest form of physical marker is a barcode, which is well known from shopping experiences in supermarkets. This type of 2-dimensional (2D) or 3-dimensional (3D) barcode, first used in 1974, is globally standardized by the GS1 organization and widely used [34]. At the points of sale, information in the barcode is compared with a static database. As a more sophisticated marker system, radio-frequency identification (RFID) chips can be attached to a good or material that carries information passively throughout the value chain with easy and independent accessibility [35]. As a drawback, RFID materials, mainly silicon and various metals, will act as contaminants in recycling processes, which need to be removed and can create operational problems in recycling facilities [35].

Besides the literature, initiatives exist in the industry that concentrate on physical tracking in plastics recycling. The industry-driven initiative on digital watermarks “HolyGrail 2.0” was founded with the aim to develop digital watermarking technologies as markers for the more accurate sorting of packaging material and to prove its applicability in large-scale trials across Europe [36]. Digital watermarks are invisible optical codes at the size of a postage stamp that can carry a wide range of product attributes and can be identified in a sorting plant via optical sensors.

In conclusion, a broad range of advantages are related to the introduction of marker technologies in plastics recycling. However, a common drawback of all marker technologies is the installation of additional equipment within sorting facilities and the establishment of databases to retrieve information from marker systems.

A physical tracer is characterized by the addition of a specific material to a polymer matrix, surface, or label, which can be identified at any point in time with the use of a special light source or device. In research and industry, many different approaches are known. The first works on the development of tracer systems in academia started in 1992 [38]. Arenas-Vivo et al. [39] studied the incorporation of the V-Quinn tracer at a concentration of up to 10^−3^ wt% in PE-HD material and ultraviolet (UV) lamps as a light source. V-Quinn is a UV fluorescence quinacridone color, modified with C14-alkane groups to ensure incorporation in the polymer matrix. Maris et al. [40] conducted studies using tracers of metal oxides doped with rare earth and rare earth metal mixtures in a concentration between 25 and 250 ppm compounded into the matrix of carbon black containing PP from the automotive industry and titanium dioxide (TiO2)-colored acrylonitrile butadiene styrene (ABS). For detection, a light source with the spectral range of 525–615 nm was used. Ahmad et al. investigated three different fluorescence tracers incorporated into PE-HD, PE-LD, PP, polyvinyl chloride (PVC), and PET [41]. UV light in a spectral range of 310–370 nm was used to identify sample bottles with 95% purity. Brunner et al. [42] added four different perylene derivates, which show fluorescence behavior in the spectral range of 450–800 nm, into the polymer matrix of polyoxymethylene (POM), polybutylene terephthalate (PBT), and acrylonitrile styrene acrylate (ASA) on the flake level. Woidasky et al. [43] investigated rare earth metals as a fluorescent physical tracer and used them in amounts of 100–500 µg/cm², [44]. Near-infrared light (NIR) (800–980 nm) was used as a light source to make tracers visible with PE-HD [43], whereas Fourier-transform infrared spectroscopy (FTIR) was chosen for PVC [44]. Bezati et al. [45] used X-ray fluorescence spectrometry (XRF) to detect various rare earth oxides in amounts of 100–1000 ppm incorporated into PP.

In addition to the scientific community, several companies and initiatives developed and investigated physical tracking by tracers. PolySecure [46] offers a commercial proprietary solution for sorting plastics based on fluorescent particles. Schmidt et al. [47] investigated the sorting efficiencies of labels and PE-HD bottles using a printed PolySecure tracer in a concentration of 100–200 µg/cm², which were identified with industrial NIR sorters. Moreover, the PolySecure technology is involved in several national and international projects related to plastics recycling. Several other initiatives are using fluorescent materials for tracking. In comparison, by using TagTec technology [48], a masterbatch with integrated taggant particles is used, where a unique identifier can be added to the polymer matrix and recognized during the sorting process. DNA brand protection is another application of a physical tracer, already commercially used for product tracing. It aims to have the possibility of securing branded products against forgery. Therefore, very small amounts of a unique tracing code are added during production in inks, additives, or labels. The retrieval of the incorporated information from the product is extensive and only comes into force in the event of customer claims. One example of DNA brand protection is DNA Smartmarks^TM^ [49], which uses a secret DNA matrix that can be retrieved in a laboratory to track branded products. Instead of DNA, sequence-defined macromolecules can also be used for chemical coding [50].

For most of the physical tracer applications, the material stays inside the polymer matrix or on the surface during and after recycling processes, which can lead to identification problems in subsequent recycling cycles and to cross-contaminations. Another drawback can arise from vast numbers of initiatives popping up such as mushrooms without alignment, and for each method, additional equipment is probably needed within sorting plants.

**Table 1:** Relevant papers and initiatives about physical tracking for plastics.

Papers about Markers for Plastics		
Aliaga C., et al.	Influence of RFID tags on recyclability of plastic packaging	[35]
Initiatives about markers for plastics		
Digital Watermarks Initiative HolyGrail 2.0	Digital watermarks for smart packaging recycling in the EU	[36,51]
Papers about tracers for plastics		
Arenas-Vivo A., et al.	Fluorescence labeling of high density polyethylene for identification and separation of selected containers in plastics waste streams	[39]
Maris E., et al.	Polymer tracer detection systems with UV fluorescence spectrometry to improve product recyclability	[40]
Maris E., et al.	Handbook of Recycling—Chapter 28—Recycling and Labeling	[26]
Ahmad S.R.	A new technology for automatic identification and sorting of plastics for recycling	[41]
Brunner S., et al.	Automated sorting of polymer flakes: Fluorescence labeling and development of a measurement system prototype	[42]
Fomin P., et al.	Investigation of fluorescence spectra disturbances influencing the classification performance of fluorescently labeled plastic flakes	[52]
Woidasky J., et al.	Inorganic fluorescent marker materials for identification of post-consumer plastic packaging	[43]
Woidasky J., et al.	Identification and Sorting of Polymers in a Circular Economy Using Fluorescent Tracer Materials	[44]
Bezati F., et al.	Addition of tracers into the polypropylene in view of automatic sorting of plastic wastes	[45]
Schmidt J., et al.	Challenges and Solutions for Plastic Packaging in a Circular Economy	[47]
Gasde J., et al.	Plastics Recycling with Tracer-Based-Sorting: Challenges of a Potential Radical Technology	[53]
Martens S., et al.	Multifunctional sequence-defined macromolecules for chemical data storage	[50]
Initiatives about tracers for plastics		
PolySecure	Solution for material traceability, product tracking and product protection by fluorescent fingerprint	[46]
TRITRACE	Detection of tracers in PP and ABS	[40]
Bir-MARK	Bio-based IR-marker for mono-material plastic recyclates	[54]
POLYMARK	EU project for development of a food container marking and identification system	[55]
Nextloopp	Creating circular food-grade recycled polypropylene from post-consumer packaging with fluorescence marker system	[56]
MaReK	Project to show use of fluorescent markers for waste management applications	[57]
Circular FoodPack	Circular packaging consortium for direct food contact applications	[58]
Demon project	Fluorescent markers for improved plastics recycling	[59]
TagTec	Addition of marker substances in masterbatch compounding	[48]
DNA Smartmarks^TM^	DNA matrix incorporated in dyes, inks or paints to tag branded products	[49]
Veriori Authentication System	Protect brand against counterfeiting with unique authentication system	[60]
StarDust Materials	Star Mark—physical marker technology for brand and liability protection	[61]

### 3.2. Blockchain for Plastics

**Figure 5:**
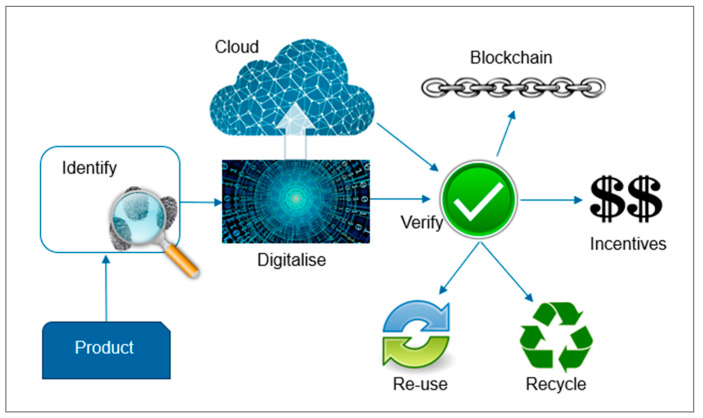
Scheme of blockchain in plastics recycling; Graphics from [37].

Blockchain is a distributed system that maintains a continuously growing list of ordered data entries and it was developed in 2008 [62]. Data are organized in blocks, with their integrity being verified through the use of digital signatures and cryptographic algorithms [63]. Considering this, blockchain offers the highest level of transparency, data integrity, and security. It is already used in well-known forms of cryptocurrencies such as Bitcoin or Ethereum. Blockchain technology is also used in pilot platforms for the food supply chain to guarantee end-to-end traceability [64]. The European Commission sees blockchain as one of the key emerging technologies for Europe and has released a blockchain strategy to shape Europe’s digital future [65]. In relation to data access, there are three main methods of participation [66]. While a public blockchain type allows everyone to participate anonymously without permission, a consortia-type blockchain accepts only access from members [67]. A third type is a private blockchain, where a private organization fulfills database management and auditing [68]. Blockchain concepts are able to offer unsurpassed transparency and secure privacy preservation on transactions at the same time [69]. Although blockchain is a relatively new concept, it shows potential and advantages for versatile use cases in many industry areas. The World Economic Forum (WEF) discovered more than 65 potential use-case solutions for blockchain in industries ranging from environmental to supply-chain applications [70]. On the other hand, high-energy consumption, false information, a lack of governance, money laundering, and inefficiency are possible obstacles related to blockchain technology [71]. Figure 5 illustrates a scheme of how blockchain could work in a circular economy for plastics.

Scientific work on blockchain technology combined with plastic and a link to the circular economy started in 2019 [72]. A Scopus search on ((plastic OR plastics) AND blockchain) resulted in 41 hits. Table 2 summarizes the most relevant papers and initiatives for blockchain in the plastics industry.

To support a circular economy in the plastics industry, several concepts have been developed independently and proposed in the literature. PlasticChain [73] favors a hybrid-plastic-product-based blockchain system that distinguishes between customer and manufacturer information, which is represented in separate blocks. PlasticCoin [74], on the other hand, is a circular economy and plastic reuse community relying on an eco-friendly consortium based on Hyperledger blockchain technology, where user allocations are settled in the blockchain instead of smart contracts. PlasticBank [75] uses blockchain technology to track and trace collected plastic waste in selected countries, reduce marine plastic pollution, and provide an additional source of income [76]. Mondal et al. [77] proposed a blockchain-based plastics recycling supply chain system that handles interaction and smart contracts at different blockchain layers for application, smart contracts, incentives, consensus, and networks. Chidepatil et al. [78] described an approach that uses multi-sensor data (i.e., visual, near-infrared, and far-infrared) within a sorting step as a starting point to create blockchain-based smart contracts with the aim of building trust for recycled plastics. 

Another trend for applying blockchain in the plastics industry is marine preservation, where start-ups generate revenue from plastic waste recovery from marine origin and increase the circular economy in the value chain [79,80]. Tsao et al. [81] proposed blockchain as a technology to remove information asymmetry and create mutual trust for marine debris recycling. Oceanworks [82] offers a commercial solution based on digitized blockchain traceability and material quality assurance for plastics sourced from oceans.

When it comes to existing initiatives, GreenToken^®^ [83] represents a commercial, private blockchain initiative for the transparent tracking of plastic material throughout the resource lifecycle. The Circularise [84] initiative developed a blockchain-technology-based software platform allowing end-to-end traceability and secure data exchange for industrial supply chains. Boiler et al. [85] investigated how the CIRbase platform of CirculaRise can contribute to the transition toward a circular economy for four different acting groups. CIRbase can help overcome barriers of a lack of data and fragmented value chain, and create trust with lower implementation hurdles for manufacturers. Security Matters [86] developed integrated blockchain-based solutions to solve both authentication as well as track-and-trace challenges within supply chains and plastics recycling. ReciChain [87] is a pilot program initiated by BASF to enable better sorting, traceability, and transparency across the value chain. It consists of a physical tracer that identifies and tracks key plastic characteristics and a blockchain marketplace that creates and translates the digital twin, providing a secure and auditable transfer-of-ownership and additionally assigns incentives.

**Table 2:** Relevant papers and initiatives about blockchain for plastics.

Papers about Blockchain for Plastics		
Cluchet P., et al.	PlasticCoin: An ERC20 implementation on Hyperledger fabric for circular economy and plastic reuse	[74]
Liu C., et al.	Plastic credit: A consortium blockchain-based plastic recyclability system	[73]
Böckel A., et al.	Blockchain for the Circular Economy: Analysis of the Research-Practice Gap	[71]
Katz D.,	Plastic bank: Launching social plastic revolution	[75]
Bhubalan K., et al.	Leveraging blockchain concepts as watermarkers of plastics for sustainable waste management in progressing circular economy	[76]
Gong Y., et al.	Blockchain application in circular marine plastic debris management	[79]
Chidepatil A., et al.	From trash to cash: How blockchain and multi-sensor-driven artificial intelligence can transform circular economy of plastic waste?	[78]
Sankaran K.,	Carbon emission and plastic pollution: How circular economy, blockchain, and artificial intelligence support energy transition?	[88]
Kahya A., et al.	Blockchain-enabled Personalized Incentives for Sustainable Behavior in Smart Cities	[89]
Khadke S., et al.	Efficient plastic recycling and remolding circular economy using the technology of trust–blockchain	[90]
Hristova T.	The place of the blockchain in the recycling of raw materials	[91]
Tsao T.J.	The Economic Theoretical Implications of Blockchain and Its Application in Marine Debris Removal	[81]
Mondal S., et al.	A Blockchain Based Transparent Framework for Plastic Waste Management	[77]
Bolier M., et al.	Accelerating the transition towards circular economy within the built environment	[85]
Dijkstra H. P., et al.	Marine plastic entrepreneurship; Exploring drivers, barriers and value creation in the blue economy	[80]
Initiatives about blockchain for plastics		
Security Matters	Digitising physical objects on the blockchain to enable the circular and closed loop economy	[86]
ReciChain	Pilot blockchain initiative for recycling of plastics	[87]
Oceanworks	Digitized blockchain-based traceability, material quality assurance for plastics sourced from oceans	[82]
GreenToken	Private blockchain technology to make the supply chain transparent and track the process of plastic materials throughout the resource lifecycle	[83]
PANDA	Plastic packaging data space—Project proposal to set up and test a circular economy data space focused on plastic packaging	[92]
ChemChain	Blockchain platform to transfer information on chemicals in products along the value chain	[93]
Plastiks	Global utility NFT marketplace to support plastic recovery around the world	[94]
TrackCycle	Blockchain based track & trace solution for hard-to-recycle plastics	[95]
SecureMarkingTM	Secure end-to-end supply chain security platform based on blockchain technology	[96]
Circularise	Blockchain solution to increase traceability and ensure privacy in supply chain	[84]
KleanLoop	DApp Blockchain SAS platform for end-to-end tracking of recycling, waste processing and waste-to-energy	[97]
IOTA Foundation	Distributed ledger technology enabling secure exchange of both value and data	[98]

### 3.3. Digital Product Passport for Plastics

**Figure 6:**
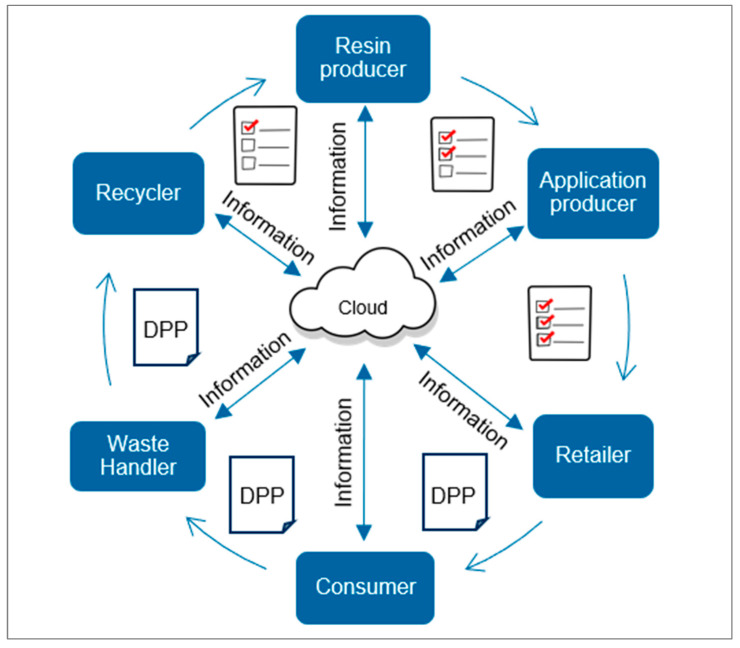
Scheme of digital product passports in plastics recycling; Graphics from [37].

The digital product passport (DPP) means the combination of a unique product identifier and the collection of data along the value chain from material to product with the aim of creating a connection between product and data [99]. Access to data, flexibility, transparency, and the accountability of customers are promised advantages of DPP solutions [100]. On the other hand, challenges regarding diverging interests of stakeholder groups, missing underlying standards, missing infrastructure, and leakage of individual know-how and intellectual property rights (IPRs) still remain [100]. Privately owned and maintained databases for DPP solutions are another obstacle and a potential source of leakage and forgery. Figure 6 illustrates a scheme about how a digital product passport could work in a circular economy for plastics. Scientific publications on digital product passports combined with plastic and a link to the circular economy started in 2014. A Scopus search on ((plastic OR plastics) AND digital passport) resulted in 20 hits. Table 3 summarizes the most relevant papers and initiatives for digital product passports in the plastics industry. 

Intentions for realizing DPPs are included in the EU Circular Economy Action Plan 2020 and are part of the Ecodesign for Sustainable Products Regulation (ESPR) [101]. King et al. [102] interpreted this European ambition on DPPs as a nine-level, systematic model for a circular economy, providing information across multiple product life cycles and across borders. 

Plociennik et al. [103] proposed a cloud-based Digital Lifecycle Passport (DLCP), filled with data during production processes, to enable a better recycling process and generate a robust lifecycle assessment. Hsu et al. [104] identified a lack of traceability, unclear target setting, missing standards, and a lack of understanding and knowledge across the value chain as key barriers associated with information-based systems in the plastic circular economy.

Ellsworth-Krebs et al. [105] proposed a mandatory digital passport with an open data standard for all reusable plastic packaging products. A standardized digital passport can overcome mistrust from cooperates and can lead to the simple transfer of data from manufacturers, brand owners, retailers, or regulatory requirements. However, the misuse of shared data, geographical limitations, and legislation are listed as critical points. 

Due to the intention from politics and the industry, several initiatives are concentrating on realizing DPPs in plastics recycling. The cross-industry consortia R-Cycle [106] collects all recycling-relevant information based on the open GS1 standard and summarizes them on a common platform with the aim to improve sorting and recycling processes for plastics. SmartID [107] favors the idea of upgrading the classical barcode system and integrating product information and security requirements as new features that are available offline. The main limitation factors for industry initiatives on digital product passports are data ownership, a lack of transparency, unclear business models, and missing standards.

**Table 3:** Relevant papers and initiatives about digital product passports for plastics.

Papers about Digital Product Passports for Plastics		
King M. R. N., et al.	A proposed universal definition of a Digital Product Passport Ecosystem (DPPE)	[102]
Plociennik C., et al.	Towards a Digital Lifecycle Passport for the Circular Economy	[103]
Hsu W.-T., et al.	Closing the loop on plastics in Europe: The role of data, information and knowledge	[104]
Ellsworth-Krebs K., et al.	Circular economy infrastructure: Why we need track and trace for reusable packaging	[105]
Initiatives about digital product passports for plastics		
R-Cycle	Recycling of plastics with the open digital standard R-Cycle	[106]
SmartID	Unique and counterfeit-proof authentification system for products	[107]
IOTA DPP 2.0	Digital Product Passport initiative based on IOTA Foundation technology	[108]
DiGiDo	Digital platform for B2B data exchange in plastic industry	[109]

### 3.4. Certification Systems for Plastics

**Figure 7:**
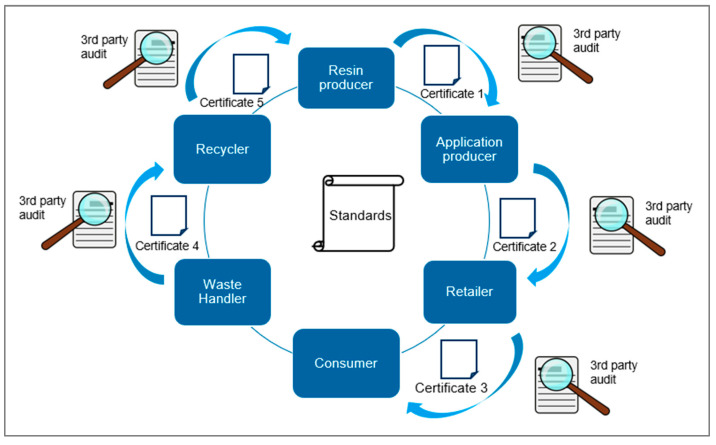
Scheme of certification in plastics recycling; Graphics from [37].

Standards and labels identifying products as recyclable have been established since the 1970s with the famous Mobius Loop, commonly composed of three arrows [110]. For plastics, the resin identification code (RIC) is widely used to distinguish different polymer resins during their life cycle and recycling. Besides the many standards for polymers, the traceability of polymeric materials has only recently become a topic covered by standards [111]. A lot of alignment within standardization organizations is needed during the development of standards, which makes publication and implementation slow. Certification systems ensure compliance with standards, rules, and regulations. Operability is frequently checked with internal and external audits, whereas the system is not checked in single compliance cases, which opens the door for misuse and forgery. There are plenty of very general or application-specific certification systems available. This paper focuses on certification systems that supports the circular economy and the tracking of plastics. For scientific literature on certification, a Scopus search on ((plastic OR plastics) AND (certification)) delivered 498 hits when results were limited to engineering, chemistry, and material science. Figure 7 shows a scheme for how certification and standards could work in a circular economy for plastic materials. For such certification systems and to enable trustworthy implementation, regular auditing is required. Table 4 lists all relevant papers, standards, and initiatives for plastics to support the circular economy. 

Shamsuyeva et al. [110] investigated standardization in the recycling industry and identified a lack of international recycling standards in a regionally organized waste management system as a main requirement for a circular economy. Nazareth et al. [112] proposed a clear governmental framework for bio-based, bio-degradable, and compostable polymers to avoid greenwashing, inform customers, and ensure proper end-of-life management. Involvement of the right stakeholders was identified as a main obstacle, whereas the education of consumers and setting the basis for decisions are seen as the main advantages of a widely accepted framework. ISCC PLUS [113] is a widely used global certification system that offers certifications for environmentally, socially, and economically sustainable production and promotes traceability throughout supply chains. RecyClass [114] is a fact-based certification and labeling system that verifies recyclability, recycled content claims, and designs for recycling guidelines. REDcert² [115] offers certification schemes for industrial production to secure sourcing from sustainable agricultural raw materials. It is important to consider that sustainable procurement does not necessarily mean that there is no competition with the food chain. EuCertPlast [116] offers a certification scheme focusing on the traceability and quality of recycled materials in end-products, with a focus on recyclers and the recycling process. CircularAssure [117] provides a risk-based quality assurance program with testing and certification that can be applied at waste handlers, chemical recycling companies, polymer producers, or brands.

Certification systems and standards can contribute to realizing a true circular economy by increasing trust within transactions from one player in the value chain to another. Besides the basic information on plastic material and parts, more specific requirements and more detailed data are necessary depending on the targeted application. A realistic approach to what is needed in which situation is a main factor for the success of information-based systems. The drawbacks of certification systems and standards are that there are too many initiatives with different targets; no traceability on the object level is possible; they do not cover all aspects; there are participants across the plastic value chain, which has to be harmonized to enable broad acceptance. 

**Table 4:** Relevant papers, standards, and initiatives about certification systems for plastics.

Papers and Standards about Certification Systems for Plastics		
Shamsuyeva M., et al.	Plastics in the context of the circular economy and sustainable plastics recycling	[110]
Nazareth M. C., et al.	Key issues for bio-based, biodegradable and compostable plastics governance	[112]
EN 15343	Plastics recycling traceability and assessment of conformity and recycled content	[111]
ISO/IEC 15459	Information technology—Automatic identification and data capture techniques	[118]
EN ISO 14021	Environmental labels and declarations—Self-declared environmental claims	[119]
DIN SPEC 91446	Classification of recycled plastics by Data Quality Levels for use and (digital) trading	[120]
Initiatives about certification systems for plastics		
ISCC PLUS	Certification system addressing sustainability requirements for all feedstocks and markets	[113]
RecyClass	Certification and labelling system for verification of recyclability, recycled content claims and design for recycling guidelines	[114]
REDcert²	REDcert² offers certification schemes for sustainable agricultural raw materials for the chemical industry	[115]
EuCertPlast	European certification system for plastics recycling	[116]
SuCCESS	Castor farming certification for bio-based polymeric material	[121]
PolyCert Europe	Recycled content quality system verification scheme	[122]
CircularAssure	Risk-based quality assurance program for plastics with assurance, testing, certification services	[117]
Empower.eco	Platform for plastic credits and tracking of plastic materials	[123]
PlastiLoop	Closed trade platform with full traceability	[124]
PlasticFinder	Certified circular plastics trading platform	[125]

## 4. Conclusions

Currently, it appears that plastics recycling is only skimming the low-hanging fruit of the plastics industry. To reach intended recycling targets, a greater effort and disruptive innovations are necessary. Information-based solutions for the physical or digital tracking of plastics can support the circular economy of plastics, help overcome hurdles, and increase recycling. Furthermore, due to the availability of data, it enables specific applications for which the origin of the used materials is relevant (e.g., food contact materials). Many proposals and developments are currently available. Nevertheless, the all-in-one solution has not been found yet. In the end, a combination of the smartest physical and digital tracking technologies could be the solution to sustainably establishing material tracking in various applications in the market.

**Figure 8:**
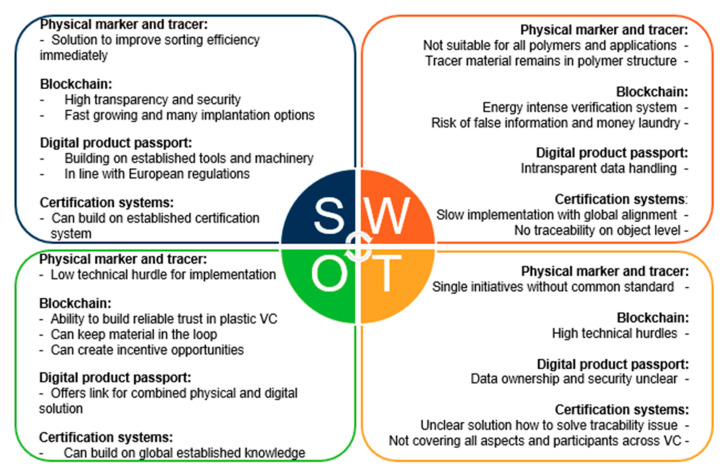
Summary of the SWOT analyses on physical and digital tracking solutions in plastics recycling.

To identify strengths, weaknesses, opportunities, and threats, a SWOT analysis was performed for each tracking technology presented above. Figure 8 shows a summary of the SWOT analyses for all four tracking technologies.

Physical tracking systems can improve sorting efficiencies on short notice. However, for each polymer type and application, a different solution is currently being developed. Blockchain technologies, as one example of a digital tracking system, have the power to establish trust and transparency in the circular economy of plastics. Nevertheless, technical hurdles and the high amount of energy that is needed for the verification systems represent obstacles for these technologies. The digital product passport is a new development that combines physical and digital tracing into one solution, but it lacks transparency in data handling and information security. Certification systems are globally established but very slow in implementation. This, together with the vagueness of how to solve the traceability issue, led to resistance to a homogenized realization of certification system solutions. In the end, the result could be a combined solution that is able to keep plastics in the loop and is accepted by all players in the plastics industry value chain and society in general.

## Data Availability

The data presented in this study are available on request from the corresponding author.
